# Supplementary material for: Early epidemiological assessment of the transmission potential and virulence of coronavirus disease 2019 (COVID-19) in Wuhan City, China, January–February, 2020
Source: BMC Med. 2020 Jul 15;18:217. doi: 10.1186/s12916-020-01691-x (PMC7360469; doi:10.1186/s12916-020-01691-x)
Supplement: Supplementary file 1 — Additional file 1 : Table S1. Information related to Japanese evacuees from Wuhan City on board government–chartered flights. Table S2. Main differences between our study and previous study. [file 12916_2020_1691_MOESM1_ESM.docx]

# Additional file 1:

# Early epidemiological assessment of the transmission potential and virulence of coronavirus disease 2019 (COVID-19) in Wuhan City: China, January-February, 2020

1. Appendix Tables S1
2. Appendix Tables S2

Table S1. Information related to Japanese evacuees from Wuhan City on board government-chartered flights

| Date of departure | Number of passengers | Number of confirmed cases |
| --- | --- | --- |
| January 29, 2020 | 206 | 4 |
| January 30, 2020 | 210 | 4 |
| January 31, 2020 | 149 | 3 |
| February 7, 2020 | 198 | 1 |

Table S2. Main differences between our study and previous study Information related to Japanese evacuees from Wuhan City on board government-chartered flights

|  | Mizumoto, et al. | Verity, et al.[1] |
| --- | --- | --- |
| IFR estimate | 0.12% (0.08-0.17%) | 0.657% (0.389–1.33) for over all |
| Age adjusted | No | Yes |
| Modeling |  |  |
| Assumption |  | - Assuming a constant attack rate by age and adjusting for demography |
| Joint estimation | Joint estimation with reproduction number | - Joint estimation with age-stratified CFR^a^ - For CFR estimation, age-based and location-based under-ascertainment were incorporated |
| Epidemic growth | Renewal equation | Exponentially growth |
| Growth rate adjustment | Yes. The data of repatriation flights were incorporated. | Yes. The data of repatriation flights were incorporated. |
| Dataset |  |  |
| Death | - Reported deaths in Wuhan City prior to Feb 11, 2020 | - Reported deaths in Wuhan prior to Feb 11, 2020 (not age stratified) - Reported death in mainland Chana prior to Feb 11 (age stratified) |
| Infected | - 4 flights (1 country) that departed between Jan 29 and Feb 7   - 4/206 on Jan 29   - 4/210 on Jan 30   - 3/149 on Jan 31   - 1/198 on Feb 7   (Total: 12/763)  (Positive/Number tested)   - No. of time points to estimate : 4 | - 6 flights (5 countries) that departed between Jan 30 and Feb 1, 2020 - 6/689 (Positive/Number tested) - No. of time points to estimate : 1 (Jan 31) |
| Population | - Population in Wuhan - 10,000,000 | - Population in Wuhan - 11,081,000 |

^a^ CFR: case fatality ratio

Reference

[1] Verity R, Okell LC, Dorigatti I, Winskill P, Whittaker C, Imai N, et al. Estimates of the severity of coronavirus disease 2019: a model-based analysis. Lancet Infect Dis. 2020. pii: S1473-3099(20)30243-7
